# Super-resolution imaging in live cells

**DOI:** 10.1016/j.ydbio.2014.11.025

**Published:** 2015-05-01

**Authors:** Susan Cox

**Affiliations:** Randall Division of Cell and Molecular Biophysics, King׳s College London, SE1 1UL, UK

**Keywords:** Super-resolution microscopy, Stimulated emission depletion microscopy, Structured illumination microscopy, Localisation microscopy

## Abstract

Over the last twenty years super-resolution fluorescence microscopy has gone from proof-of-concept experiments to commercial systems being available in many labs, improving the resolution achievable by up to a factor of 10 or more. There are three major approaches to super-resolution, stimulated emission depletion microscopy, structured illumination microscopy, and localisation microscopy, which have all produced stunning images of cellular structures. A major current challenge is optimising performance of each technique so that the same sort of data can be routinely taken in live cells. There are several major challenges, particularly phototoxicity and the speed with which images of whole cells, or groups of cells, can be acquired. In this review we discuss the various approaches which can be successfully used in live cells, the tradeoffs in resolution, speed, and ease of implementation which one must make for each approach, and the quality of results that one might expect from each technique.

## Highlights

• Super-resolution imaging of cell structures can achieve a resolution of tens of nm.
• There are three major techniques: STED, SIM, and localisation microscopy.
• Live cell super-resolution requires trading off resolution, speed, and light dose.

## Introduction

**Fig. 1:**
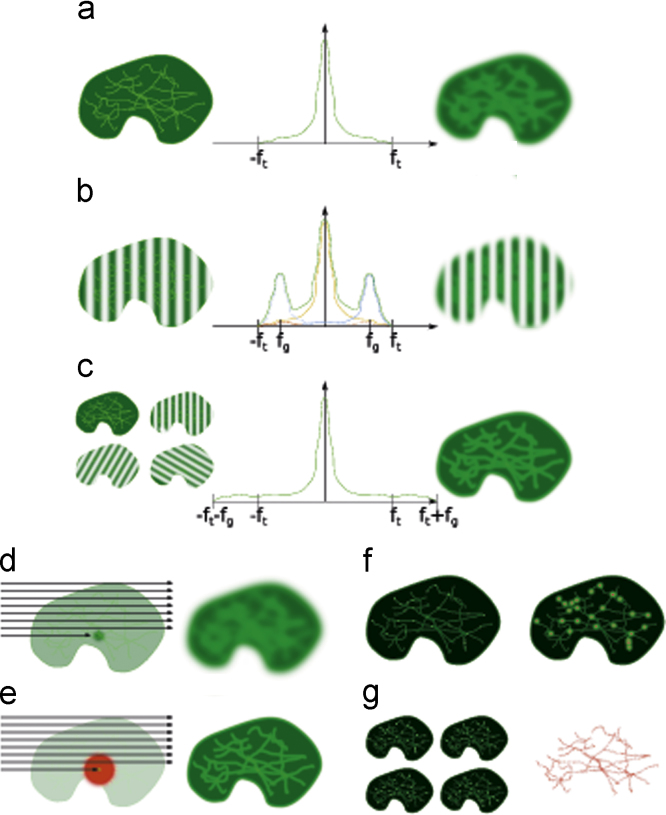
Fluorescence super-resolution methods. (a) Structured illumination microscopy. When a sample (left) is imaged, the resultant image is convolved with the point spread function of the microscope (right). This image has information at a range of frequencies, up to the transmission frequency of the microscope (middle). (b) When a grating is projected onto the sample, then in frequency space the sample information is reproduced at the grating frequency (blue). Due to this shift in frequency, frequencies not visible in the widefield image are shifted into the visible range (red). The resulting image (green) is the sum of the original image (yellow), and the positive (blue/red) and negative (dotted) frequency shifted copies. (c) By taking multiple images at different angles, the shifted higher frequency information can be extracted, giving a super-resolution image. (d) Stimulated emission depletion microscopy. In confocal microscopy, a diffraction limited point of light is scanned across the sample (left) giving a diffraction limited image (right). (e) If a doughnut shaped depletion beam is used, the effective beam size is smaller (left) and so the image from naturally emitted light is sharper (right). (f) Localisation microscopy. Sparse sets of fluorophores are excited (left) and imaged (right). The centers of the diffraction limited spots are localised (red). (g) By repeating this process many times (left) a super-resolution image of the sample can be built up from these localised centres (right). (For interpretation of the references to color in this figure caption, the reader is referred to the web version of this paper.)

Fluorescence imaging is a ubiquitous tool in cell biology and biomedical research. It is extremely useful because it allows the dynamics of different proteins to be observed in live cells. The resolution of the images is limited by the microscope and the wavelength of the light used. The wavelength of light is generally in the visible region; shorter wavelengths offer better resolution, but as the wavelength shortens to ultra-violet, the light becomes toxic to cells. For achieving high resolution, good quality optics with an objective designed to work with a high refractive index medium such as oil is essential. However, even with all these optimisations, the resolution of a standard fluorescence system is limited by the wave properties of light to around 200 nm (see Fig. 1a).

Until about ten years ago, imaging of structures smaller than this had to be carried out either in the near field, most commonly using total internal reflection microscopy (which is limited to imaging structures within about 100 nm of the coverslip), or by electron microscopy, which can only be carried out in dead cells and requires significant sample processing. Over the last twenty years, three major super-resolution fluorescence techniques have been developed which break this limit and allow imaging at a lengthscale down to tens of nm (Heintzmann and Ficz, 2007; Hell, 2009). The first approach, stimulated emission depletion microscopy (STED) (Klar et al., 2000), is based on a confocal microscope, in which a diffraction limited spot is scanned over the sample. Super-resolution is achieved by shrinking the area from which light is detected. The second approach, structured illumination microscopy (SIM), projects patterned illumination onto the sample to downshift high frequency information and allow it to be recorded (Gustaffson, 2000; Gustaffson et al., 2008). Localisation microscopy, also known as photoactivatable localisation microscopy (PALM) (Betzig et al., 2006), fluorescence photoactivation localisation microscopy (fPALM) (Hess et al., 2006), and stochastic optical reconstruction microscopy (STORM) (Rust et al., 2006), among other names, exploits the fact that the position of a feature of a known shape can be found with a precision not limited by the size of the shape. Instead of taking an image of the sample, a composite image is built up by finding the positions of many individual fluorophores to very high precision.

Accessing these methods involves either buying a commercial system or building a custom system. The difficulty in doing so varies considerably with the technique; the simplest localisation microscopy measurements require only a high quality widefield microscope and powerful laser illumination, with the complexity of the setup increasing if more control over fluorophore behaviour, or three dimensional imaging is required. SIM and STED require an exactly aligned setup with a sophisticated control system and constructing one is outside the reach and interests of most biological labs. Because of the expense and time involved in making super-resolution measurements, it is critical to consider which technique is most suitable to answer a particular biological question. In this review, we will consider the different properties of each of the techniques and what measurements this might make them particularly suited for, with particular reference to achieving super-resolution in live cell samples.

## What is resolution?

The resolution of a system can be defined in a number of different ways. A common approach to defining resolution is to ask how close together two point sources can be, and still allow an observer to know that the resulting image arises from two point sources rather than one, and find the positions of those sources.

Obviously, if a point source of light was imaged to a point, the answer would be infinitely close. However, the resolution of light microscopy is ultimately limited by the wave-like properties of light. Photons interfere with each other, and with themselves, which means that a point source of light cannot be focused back to a point; the best that can be achieved is a spot with a size proportional to the wavelength of the light. In an actual fluorescence microscope, the resolution will also be limited by the range of angles at which light can pass into the objective (determined by the numerical aperture). These factors mean that a point source gives rise to an Airy disk when imaged by a microscope (the point spread function).

There are a number of criteria which can be used to calculate the resolution given these restrictions, for example the Rayleigh criterion and the Abbe limit, all of which give similar answers. The variation is due to different definitions of how low the dip between two point spread function shapes has to be before one can confidently classify the observed image as arising from two separate point sources.

A very helpful concept when considering resolution is that of spatial frequency. Any image can be considered as being built up of signals of different frequencies and amplitudes. A blurry image (e.g. one out of focus) will contain only low spatial frequencies. An image with a sharper appearance (e.g. with well defined edges) will contain higher spatial frequencies. The highest spatial frequency that an objective can transmit (which can be considered in real space as the range of angles it transmits) thus determines its resolution. Of course, all of these ideas assume an imaging system with well defined spatial frequency transmission characteristics forming an image of a sample; an assumption that will not necessarily hold for all techniques.

Thinking of the imaging system in terms of point spread functions and in terms of spatial resolution gives us two possible approaches to achieving resolution below the Abbe limit. We can change the range of frequencies that are transmitted through the imaging system, or we can learn where in space the point sources are with greater accuracy.

## Structured illumination microscopy

SIM makes higher frequency information available in the image by illuminating the sample with patterned light. A fine grating is placed in an intermediate image plane of a widefield microscope, or is created using a spatial light modulator (Gustaffson, 2000; Gustaffson et al., 2008; Heintzmann, 2003). This pattern is transmitted through the optical system, and projected onto the sample. The grating is at a single defined spatial frequency. The recorded image is an interference pattern between this grating and the sample (see Fig. 1b). Because of this interference, high frequency image information is shifted down to lower frequencies. This means that information at spatial frequencies that were previously too high to be passed through the optical system can now be transmitted. By taking multiple images (usually nine or fifteen, that is three or five phase positions over three angles) and analysing them, a reconstructed image containing the high-frequency information shifted back to their original frequencies can be produced (see Fig. 1c). The resolution of this image is determined by the highest spatial frequency present in it, which in turn is determined by the frequency of the grating. Since the maximum frequency of the grating is limited by the maximum frequency that the objective can transmit, the best resolution boost structured illumination can give is a factor of two.

If a grating which only varies in *x* and *y* is used, there will only be an in-plane resolution improvement. Alternatively, a modulation of the excitation light in the axial direction can be created using three beam interference which improves the *z* resolution by a factor of two (Gustaffson et al., 2008; Schermelleh et al., 2008). Generally, achieving two dimensional resolution improvement requires nine images, whereas three dimensional improvement requires fifteen. It is possible to improve on this using non-linear methods (linear means that intensity in is proportional to intensity out, which is no longer true when the sample saturates), which use saturation to create illumination patterns that contain higher frequencies than the base frequency (Heintzmann et al., 2002; Gustafsson, 2005). However, while this method has been demonstrated in cells (Rego et al., 2012), it is highly challenging and not currently available commercially.

Since structured illumination microscopy only requires nine or fifteen widefield images to be taken to reconstruct one super-resolution image, it is well suited to live cell imaging. The only restrictions are that there should not be substantial bleaching or movement during the course of the acquisition. To image fast processes this may require very short exposures, and higher laser power to achieve a similar signal strength, in which case phototoxicity may become a concern. A further caveat is that in common with all deconvolution-based techniques, the image will have artefacts at lengthscales close to the resolution limit; interpretation of fine scale structure in the images must be done with care (for an example of a protocol which allows quantification of SIM images, see Baddeley et al., 2007). Having said this, structured illumination is probably the super-resolution technique most amenable to adaptation to live cell imaging, and it is possible to acquire the data for a single reconstructed super-resolution image in a time between a few seconds and 0.1 s depending on the system (Hirvonen et al., 2009; Kner et al., 2009).

Due to the potentially fast acquisition rate, and the fact that it can be used with any fluorophores, structured illumination has been a popular choice for live cell imaging. Two particularly interesting recent examples are imaging the DNA double strand break repair process (Lesterlin et al., 2014), and monitoring the 3D shape of the cytoskeleton in adherent cells, allowing the role of the actin arcs in flattening cells to be investigated (Burnette et al., 2014).

The major disadvantage of the SIM described above is that the method fails if there is too much out-of-focus light present, since it is not then possible to image the grating well enough to get useful information. An interesting alternative approach to SIM scans the sample with multiple diffraction limited beams and then processes the resultant image (either with image analysis or optical components). This method can achieve a resolution a factor of $\sqrt{2}$ better than widefield (diffraction limited) microscopy, giving a resolution of around 150 nm (York et al., 2012, 2013; Sheppard et al., 2013). Since the processing can be done with optical elements, this allows imaging up to 100 Hz, with results being demonstrated in live zebrafish embryos (York et al., 2013).

## Stimulated emission depletion microscopy

Stimulated emission depletion microscopy (STED) (Hell and Wichmann, 1994) also transmits higher frequency information through the microscope system, but using a completely different technique to structured illumination microscopy. It is based on a confocal microscope system, in which a diffraction limited point of light is scanned across the sample (see Fig. 1d). In a confocal microscope the resolution of the system is determined by the size of the spot when the light is focused on the sample, and by the size of the pinhole that is used to reject out of focus light.

Stimulated emission is the process whereby an excited molecule or atom is stimulated with a photon redshifted from the frequency at which it would naturally emit. The molecule or atom returns to its ground state and emits a photon with the same wavelength as the stimulating photon.

In stimulated emission depletion microscopy a doughnut shaped beam is created using light which is red-shifted from the natural emission wavelength of the fluorophore under observation (see Fig. 1e). This means that excited molecules which are illuminated by the doughnut shaped beam will emit light at a different wavelength (the depletion beam wavelength) to the molecules in the centre of the doughnut (which will emit at the natural emission wavelength). These two different sources of light can be separated using filters in continuous wave systems (Willig et al., 2007), while the discrimination and thus resolution can be improved by using timing information in pulsed systems (Hell and Wichmann, 1994; Klar et al., 2000) and lifetime information (Vicidomini et al., 2011; Moffitt et al., 2011). Because the non-depleted area is now smaller than the diffraction limited spot size, higher spatial frequency information is being transmitted through the system and the resolution is improved (see Fig. 1e). The major experimental challenge in STED microscopy is creating the doughnut-shaped depletion beam and exactly aligning it to the excitation beam. This can be avoided by using diffraction-limited excitation and depletion beams of the same shape, and using lifetime differences to achieve super-resolution (Marsh et al., 2014), simplifying the experimental setup at the cost of degrading the achievable resolution to about 100 nm.

A doughnut-shaped beam produces an improvement in the in-plane resolution but not in the axial direction. Two different approaches have been taken to achieve improved axial resolution in STED: combining it with 4pi (using two interfering beams), achieving a resolution of 33 nm in the axial direction and 250 nm in-plane (Dyba and Hell, 2002), or, as is more common, by using a different phase pattern to shape the depletion beam to give an almost isotropic resolution of 90 nm (Klar et al., 2000).

By saturating the depletion beam, the area in the centre which is not depleted shrinks. This non-linearity is key to the fact that there is no theoretical limit on the resolution of STED. The resolution is dependent on the depletion intensity, which determines how much of the excitation point spread function can be depleted, and on the excitation intensity, which determines the signal level. In a living sample, of course, there is a limit to how high an intensity can be used without damaging the sample. In live cells there is a tradeoff between resolution, speed, and the area which can be imaged, since this is a scanning technique and so speed is inversely proportional to area. STED has been demonstrated in live cells with acquisition times of a few seconds using standard fluorescent proteins (Rankin et al., 2011), achieving an in-plane resolution below 60 nm, and at video rate using organic dyes (acquiring a 1.8 μm×2.5 μm area and achieving an in-plane resolution of around 60 nm) (Westphal et al., 2008). Imaging at video rate required using an intensity of 400 MW/cm^2^ at the sample.

For biological applications, it is extremely important to be able to take images in two colour channels. In STED this can be done using two standard dyes or proteins, but this required two excitation beams and two depletion lasers (Donnert et al., 2007), making the alignment of the system challenging. An alternative approach is to use one standard dye and a second with a long Stokes shift (the difference between the absorption and emission wavelengths), so that the same depletion laser can be used for both (Pellett et al., 2011).

In order to penetrate further into living cells, two photon microscopy can be combined with STED (Moneron and Hell, 2009), allowing super-resolution live cell data to be taken up to 30 μm into tissue (Takasaki et al., 2013). However, when attempting to achieve super-resolution at these depths, the problem of aberration has to be considered. In a perfect sample the shape of the depletion beam would be transmitted without distortion, but in a biological sample there are structures with different refractive indices and aberration is inevitable, and will degrade the resolution. The degradation will increase the deeper into the sample measurements are made. It is possible to correct such aberrations using a micromirror device, and the principle has been demonstrated in fixed cell samples (Gould et al., 2012; Lenz et al., 2014). This approach promises to improve the resolution and the signal quality of STED in scattering tissue, making it more useful for whole-organism imaging. It is likely that aberrations also degrade the resolution of structured illumination images (by distorting the raw images), but experiments have only been carried out for systems that do not achieve super-resolution (Débarre et al., 2008).

## Localisation microscopy

There are a number of different localisation-based techniques, but all rely on the same basic principle: if you know something about your image you can build that knowledge into a model, perform a fit, and so create a new image with improved resolution. Localisation microscopy exploits the fact that if you know you have a single point source emitting, you can fit the position of that emitter with a Gaussian or a Lorentzian function, to an accuracy which is determined by the number of photons emitted by a molecule, the size of the point spread function, and the background (Thompson et al., 2002; Ober et al., 2004). This accuracy can be degraded by aberrations which distort the point spread function when imaging thicker samples, though these effects can be corrected to some extent using adaptive optics (Quirin et al., 2012; Izeddin et al., 2012; McGorty et al., 2014). Of course, in order for the assumption that you have a point source to be valid, there must be only a small number of fluorophores emitting in a single frame—few enough that the probability of overlap is very small (see Fig. 1f). The super-resolved image is built up from the localised positions of the individual fluorophores (see Fig. 1g), typically requiring tens to hundreds of thousands of localisations. This means that thousands or tens of thousands of frames are usually needed to build up a reconstructed super-resolution image, with each frame only allowing a few fluorophores to be localised. Separating the fluorophore emission in time in this way is achieved by switching the flurophores between a state in which they can emit light, and a state in which they cannot. Localisation microscopy can be performed with a widefield microscope, which, along with the availability of free analysis software (Henriques et al., 2010), makes this method highly accessible.

This requirement for the fluorophore emission to be well separated in time requires a high degree of control over fluorophore behaviour. This can be achieved in several ways. The first method is to use photo-activatable proteins, as in photoactivatable localisation microscopy (PALM) (Betzig et al., 2006) and fluorescent photoactivation localisation microscopy (fPALM) (Hess et al., 2006); these have the advantage that fluorescent proteins are already commonly used in live cells, and this method has been used to image focal adhesions in live cells (Shroff et al., 2008). Alternatively, conjugated dyes can be used, in which one dye activates the pair and the other is used as a readout channel, as used in stochastic optical localisation microscopy (STORM) (Rust et al., 2006). Since the same dye can be used as a readout, with different dyes conjugated for activation, this method can do two colour measurements without any chromatic aberration (though cross-talk must be taken into account when evaluating the images) (Bates et al., 2007). Alternatively, organic dyes such as Alexa647 can be used as a label, and a reducing medium and high laser power can be used to control the blinking dynamics, to achieve a suitable number of molecules in each frame, as in direct STORM (dSTORM) (Heilemann et al., 2008, 2009). This method has also been shown to work in live cells samples (Wombacher et al., 2010), though this does of course require introducing a dye into a live sample.

The main limitation of localisation microscopy when imaging in live cells is the speed of acquisition. Acquiring up to ten thousand frames typically takes at least several minutes, and many processes in cells take place on a much shorter timescale than this. However, over recent years a number of advances have been made which promise to improve imaging of dynamic systems at super-resolution. The advances have come on three main fronts: brighter probes, particularly in the infra-red, mean that the cell does not have to be illuminated for as long to record the same number of photons; building microscope systems with rapid, self-adjusting control of the lasers to enable optimised switching and excitation of fluorophores at very short timescales; and the development of high-density analysis methods, which mean that fewer frames need to be acquired.

The methods described so far improve the in-plane resolution. As the point spread function changes symmetrically on either side of focus in a widefield system, it is not possible to infer the *z* position from the size of the point spread function. However, if two images of the same sample are taken simultaneously in two different focal planes, the positions of the point spread functions can be fitted in the *z* direction as well as the *xy* plane, achieving 75 nm axial resolution (Juette et al., 2008). Alternatively, one can create a point spread function which varies with *z*, either using a cylindrical lens to create astigmatism (which achieves 50–60 nm axial resolution Huang et al., 2008) or using a phase-only liquid crystal on silicon display to create a double helix shaped point spread function, which can achieve down to 20 nm axial resolution on samples bright enough to give an in-plane resolution of 10 nm (Pavani et al., 2009). Interference methods give the highest resolution in *z* (Shtengel et al., 2009), and can achieve an axial resolution of 10 nm. Their use has revealed the nanoscale vertical structure of focal adhesions (Kanchanawong et al., 2010). Airy beams can also be used to identify *z* positions, achieving isotropic resolution at the cost of localisation density (Jia et al., 2014).

Choosing the right probe is critical for a localisation microscopy experiment. Detailed protocols for a number of fluorophores have been published (Gould et al., 2009; van de Linde et al., 2011). Photoswitchable fluorescent proteins are well suited to live cell work, but organic dyes have much higher photon yields and thus better localisation accuracy (Fernandez-Suarez and Ting, 2008). Live cell structures have been imaged with organic dyes (Wombacher et al., 2010; Klein et al., 2011), but getting the organic dye into the cell remains more challenging than using a protein. However, in cases where this problem can be solved, the development of new dyes, particularly in the infrared (Lukinavicius et al., 2013), promises lower phototoxicity in live cell measurements.

For imaging fast dynamic processes, the use of high laser intensities and fast switching has allowed imaging to be carried out in around a second in live cells (Jones et al., 2011). Such imaging requires extremely fast camera frame rates. The development of sCMOS cameras has led to such high frame rates being more accessible, though it should be noted that these cameras are prone to hot pixels which cannot be used in a measurement, and have different noise characteristics that must be taken into account when analysing the data (Huang et al., 2013).

### High-density analysis methods

The methods discussed above can improve the rate at which raw data can be acquired, but the speed is always limited by the need to acquire data in which the point spread functions do not overlap. If this requirement can be lifted, fewer frames can be used to image the same number of molecules, cutting down the time required to acquire enough data to reconstruct a localisation microscopy image. When analysing images with overlapping point spread functions more information must be built into our model. We can either try to use information about the time domain, that is about how fluorophores blink and bleach, or about the spatial domain, where we could try to build in information about what overlapping fluorophores look like.

Super-resolution optical fluctuation imaging (SOFI) (Dertinger et al., 2009, 2010) builds in an assumption about the time domain that the blinking of fluorophores is uncorrelated. This method takes higher moments of the image series, which essentially sharpens the PSF by raising it to a power. The decrease in the point spread function that this generates leads to super-resolution, without any explicit model of the PSF or the underlying structure. SOFI is fast to compute, and can work with a range of fluorophores including quantum dots, organic fluorophores (when they are induced to blink) and fluorescent proteins, giving a resolution as low as 80 nm (measured in terms of ability to distinguish two adjacent features) with 72 s acquisition time for the raw data (Geissbuehler et al., 2012).

Alternatively, the fact that a fluorophore blinking or bleaching leads to a change from one frame to the next can be exploited to achieve localisation. A series of images can be taken, differences between the images are identified and modelled as arising from a fluorophore blinking or bleaching (Burnette et al., 2011; Simonson et al., 2011), giving a resolution down to 65 nm with acquisition times down to 80 s, although both values vary considerably with the sample blinking properties. These methods enable relatively easy data acquisition, since fixed samples with standard embedding can be used (although low bleaching rates and a long lifetime for the non-emitting blinking state will allow a better resolution to be achieved).

In the spatial domain, patches of the image which are too large to arise from single fluorophores can be fitted with multiple Gaussian peaks (Holden et al., 2011; Huang et al., 2011), demonstrated on series with acquisition times between 200 s and 250 s. Alternatively, a compressed sensing approach can be taken (Zhu et al., 2012), in which the generated image is assumed to be made up of a very fine, regular, and sparse grid of emitters, which is blurred by the PSF and then sampled. This approach takes 3 s to acquire the data required for a super-resolution image, reporting a resolution of 60 nm (though the resolution is defined by the density of detected fluorophores rather than the ability to separate two features, meaning it may not correspond to resolutions measured with other techniques). An alternative approach utilizes successive deconvolution of the images to improve the resolution (Mukamel et al., 2012) (resolution estimated by theory to be 25 nm for 5000 frames of data). This has the advantage that it does not make assumptions about fluorophore appearances being well separated, although it does require the fluorophores to be far enough apart for a deconvolution to be able to separate them.

It is also possible to build in both spatial and temporal information about the fluorophores into a model, an approach taken by Bayesian analysis of blinking and bleaching (3B analysis) (Cox et al., 2012). The data is modelled as arising from a number of fluorophores undergoing blinking and bleaching. A very high degree of fluorophore overlap is possible. This has the added advantage that super-resolution data can be obtained from standard fluorescent proteins, rather than photoswitchable fluorophores or organic dyes. Superresolution images can be reconstructed from 4 s of data collected from standard fluorescent proteins with a spatial resolution of 50 nm, though the analysis requires a minimum of several hours computational time. The output is not a map of individual fluorophore positions as with other localisation methods, but is a probability map showing how the likelihood of a fluorophore being present varies spatially.

### What is the resolution of my image?

When taking a super-resolution image it is always important to understand the limits of resolution and possible image artefacts. This is particularly critical for localisation microscopy, since the result of an experiment is a set of coordinates of fluorophores, which are then reconstructed into a super-resolution image. The final resolution of the reconstructed image depends on a number of factors, with the two most common limiting factors being the localisation precision and fluorophore density. The localisation precision for a single fluorophore depends on the level of background in the image and the number of photons collected from a single molecule (a commonly used approximate calculation is given in Thompson et al. (2002) and a thorough treatment is given in Ober et al. (2004)). For samples labelled using primary and secondary antibodies, it is also important to consider the offset between the protein and the fluorophore, and if extremely high resolution is required it may be necessary to use a nanobody instead (Ries et al., 2012). Movement of the sample will also degrade the resolution. Systematic movements of the whole field of view can be corrected to some extent via drift correction, but movement within a live sample will lead to motion blur. The reconstruction of the image is generally performed by convolving a Gaussian with a list of point positions, with the Gaussian width generally reflecting the number of photons from the molecule. Evaluating the effect of all these different issues is challenging, particularly for a non-specialist user. One approach is to combine estimates of all the sources of error and calculate the resolution of your system (Rees et al., 2012), though this requires quantification of factors that may not be known exactly, such as the inaccuracy introduced by antibody labelling. Alternatively, the data can be directly assessed to see at what distance it stops being correlated. This Fourier ring correlation approach (Nieuwenhuizen et al., 2013) allows a direct calculation of the resolution, without requiring detailed knowledge of the sample.

## Conclusions and outlook

The optimal super-resolution technique to use for a particular experiment depends on the in-plane and out-of-plane spatial resolution needed, the fluorophores which can be used to label the sample, whether information from a live sample is needed and the timescale of any dynamic processes if you are imaging in a live sample. In general, SIM will achieve the highest speeds, but only a factor of two improvement in resolution, requiring a power at the sample of 1–10 W/cm^2^ (Fiolka et al., 2012); STED can achieve high speeds, up to video rate for a few microns square, but the time to acquire scales with the area needed, and requires a power at the sample of 30–540 MW/cm^2^ (Westphal et al., 2008); and localisation microscopy can achieve high speeds, up to a few frames a second, at the price of more sophisticated and time-consuming analysis of the data. For a more detailed comparison see Schermelleh et al. (2010). Light dose requirements for localisation microscopy vary from around 1 kW/cm^2^ (Shroff et al., 2008) to 12 W/cm^2^ (Cox et al., 2012), with lower powers generally only possible with photoswitchable fluorophores and long acquisition times, or when analysis-intensive methods are used.

Ideally, when imaging live cells one would limit the light dose to an intensity that does not cause a physiological response. This is highly challenging in super-resolution, which tends to require a light dose many times higher than one would normally use for fluorescence imaging. The development of selective plane illumination techniques, in which only the plane of interest is illuminated with light, allows imaging to be carried out with much lower light doses than standard imaging. A combination of SIM and SPIM has been developed, though the SIM is only used for removing out-of-focus background and not for resolution improvement (Keller et al., 2010). STED has also been combined with SPIM (Friedrich et al., 2011). Combining with localisation microscopy is challenging, as the light sheets are relatively thick, but can be achieved using careful photoactivation, or high density analysis methods (Cella Zanacchi et al., 2011; Hu et al., 2013). These new developments promise to make future super-resolution techniques faster and less damaging, allowing us to probe the dynamics of cells at the nanoscale.
